# Detrimental clinical interaction between ritonavir-boosted protease inhibitors and vinblastin in HIV-infected patients with Hodgkin lymphoma

**DOI:** 10.1186/1758-2652-13-S4-P215

**Published:** 2010-11-08

**Authors:** A Cingolani, L Torti, C Pinnetti, K de Gaetano Donati1, R Murri, E Tacconelli, LM Larocca, L Teofili

**Affiliations:** 1 Institute of Infectious Diseases, Catholic University, Roma, Italy; 2 Catholic University, Hematology, Roma, Italy; 3 Catholic University, Histopathology, Roma, Italy

## Background

Pharmacokinetic interaction between Vinca alkaloids and antiretrovirals has been widely demonstrated, even though its clinical relevance is still debated. The incidence of Hodgkin's lymphoma appears to be rising in HIV-infected people, and vinblastine - containing chemotherapy regimens are widely recommended in these pts.

## Purpose of the study

To evaluate the clinical interaction between HAART regimens and vinblastine in HIV-infected patients with HL.

## Methods

Clinical charts of all HIV-infected patients followed at our center with a diagnosis of HL were reviewed. Differences in group proportions were assessed using 2 test. One way ANOVA test was used was to test for differences among independent groups. Potential risk factors for WHO III-IV neutropenia were analysed by step forward logistic regression analysis. The Hosmer and Lemeshow goodness-of-fit test was used to assess model fit. Statistical analysis was performed using the software program Intercooled Stata .

## Summary of results

From June 2002 to July 2009 sixteen patients with HL were concomitantly treated with vinblastine-containing regimens (ABVD or Stanford V) and HAART, supported by G-CSF administration. (M/F: 11/5; median CD4 cell count: 189/µl , IQR 15-459; median HIV-RNA 5.8 log10 copies/ml, IQR 2.9-6.9 ; bone marrow was involved in 50% of cases. 43% of pts were in HL stage IV. 5 out of 16 pts were on PI/r, 2 on unboosted-PI, 7 on NNRTI (6 EFV and 1 NVP) and 2 on raltegravir. Mean nadir neutrophil count (+SD) for all cycles of PCT on the same HAART regimens were 0.218+0.343x106/L for patients taking regimens containing PI/r, 0.375+0.078x 106/L in patients taking PI-unboosted and 1.560+715 x106/L in patients on non PI-based regimens (P<0.001). After controlling for CD4 cell count < 200/µl, use of zidovudine and bone marrow involvement, the use of PIs were more likely to be associated with severe grade III-IV neutropenia (OR, 34.3, 95%CI 1.9-602.4; P = 0.02; McFadden R2:0.50). The mean nadir neutrophils count was 1.350x106/L (+SD 0.800) in patients not taking RTV, 0.850x106/L (+SD 0.091) in patients taking 100 mg of RTV, and 0.047x106/L(+SD 0.050) in those taking 200 mg of RTV as boosting, respectively.

## Conclusions

The concomitant administration of vinblastine-containing chemotherapy regimens with PIs can lead to higher levels of neutropenia and in this set of patients HAART regimens containing different classes of drugs (NNRTI, integrase inhibitors) are more advisable.

